# Genetic risk factor identification for common epilepsies guided by integrative omics data analysis

**DOI:** 10.1111/epi.70021

**Published:** 2025-11-30

**Authors:** Ashwini Mushunuri, Oluyomi Adesoji, Roland Krause, Patrick May, Holger Lerche, Albert Becker, Daniela Grimm, International League Against Epilepsy Consortium on Complex Epilepsies, Michael Nothnagel, Herbert Schulz

**Affiliations:** ^1^ Department of Microgravity and Translational Regenerative Medicine, Medical Faculty, University Hospital Magdeburg Otto von Guericke University Magdeburg Germany; ^2^ Clinic for Plastic, Aesthetic, and Hand Surgery, Medical Faculty, University Hospital Magdeburg Otto von Guericke University Magdeburg Germany; ^3^ Department of Engineering Brandenburg University of Applied Sciences Brandenburg Germany; ^4^ Cologne Center for Genomics University of Cologne Cologne Germany; ^5^ University Hospital Cologne, Medical Faculty University of Cologne Cologne Germany; ^6^ Luxembourg Center for Systems Biomedicine University of Luxembourg Esch‐sur‐Alzette Luxembourg; ^7^ Department of Epileptology Eberhard Karls University Tübingen Germany; ^8^ Institute for Cellular Neurosciences II Medical Faculty, University of Bonn Bonn Germany; ^9^ Department of Biomedicine Faculty of Health, Aarhus University Aarhus Denmark

**Keywords:** epigenetics, epilepsy, genetic generalized epilepsy, GWAS, ILAE, MAGMA, transcriptomics

## Abstract

**Objective:**

Genetic generalized epilepsies (GGEs) comprise the most common genetically determined epilepsy syndromes, following a complex mode of inheritance. Although many important common and rare genetic factors causing or contributing to these epilepsies have been identified in the past decades, many features of the genetic architecture are still insufficiently understood. This study integrates genome‐wide association study (GWAS) data from the International League Against Epilepsy Consortium on Complex Epilepsies with transcriptome‐wide association studies to identify genes whose genetically regulated expression levels are associated with epilepsy.

**Methods:**

To achieve this, we used multiple computational approaches, including MAGMA, a tool for gene analysis of GWAS data, and its derivatives E‐MAGMA and H‐MAGMA, to improve gene mapping accuracy by utilizing tissue‐specific expression and chromatin interaction data. Furthermore, we developed ME‐MAGMA to incorporate methylation quantitative trait loci data, providing insights into epigenetic factors.

**Results:**

We identified a total of 897 false discovery rate‐corrected (<.05) candidates. These include voltage‐gated calcium channels, voltage‐gated potassium channels, and other genes such as *NPRL2*, *CACNB2*, and *KCNT1* associated with epilepsy pathogenesis that act as key players in neuronal communication and signaling in the brain.

**Significance:**

In this study, we propose new candidate genes to expand the dataset of potential epilepsy‐causing genes. Further research on these genes may enhance our understanding of the complex regulatory mechanisms underlying GGE and other types of epilepsy, potentially revealing targets for therapeutic intervention.

**Key points**

- A new method called ME‐MAGMA is designed to identify genes influenced by epigenetic regulators associated with epilepsy.
- A multilayered approach is used, utilizing positional, transcriptional, and epigenetic information to identify epilepsy‐associated genes.
- The genes identified through chromatin interaction profiles from both the adult and fetal datasets suggest some possible roles in developmental effects on neurological pathways.

## 1 INTRODUCTION

Epilepsy is a common neurological disorder that affects millions of people worldwide, characterized by recurring, unprovoked seizures. It can affect individuals of all ages, impacting their quality of life. Although its causes vary from brain injuries to infections or genetics, research is increasingly focusing on genetic factors to improve diagnosis, treatment, and prevention.^1^

Epilepsy is a broad term that includes a variety of disorders, which are categorized based on seizure types, age at onset, developmental status, comorbid features, and underlying etiologies. One specific type is genetic generalized epilepsy (GGE), which refers to a group of epileptic syndromes with a strong indication of a hereditary predisposition. It is characterized by generalized seizures, meaning the seizures involve bilateral hemispheric networks from the onset. In contrast, focal epilepsy (FE) is defined by seizures that originate from a specific, localized region within one cerebral hemisphere.^2^, ^3^

Genome‐wide association studies (GWAS) are a widely used genomic approach to identifying genetic variants that contribute to disease risk^3^ and allow identification of specific genomic regions that increase the risk of developing a particular phenotype or disease, providing insights into a disorder's underlying genetic architecture, including epilepsy.^3^ The largest GWAS on epilepsy so far was conducted by the International League Against Epilepsy (ILAE) Consortium on Complex Epilepsies, which performed a large‐scale transethnic meta‐analysis by combining GWAS data from populations of European, Asian, and African genetic ancestry.^4^, ^5^ In this study, 26 genome‐wide significant loci were identified, of which 19 were specific to GGE. These loci collectively implicate 29 putative causal genes, such as *CACNA2D2, KCNIP2, SCN1A*, and *RBFOX1*, underlying the genetic architecture of epilepsy.^4^, ^5^

Despite the large size of this GWAS, many features of the genetic architecture of epilepsy are still insufficiently understood. One way forward is the identification of genes whose genetically regulated expression levels are significantly associated with complex human diseases or traits,^6^ as is the primary objective of transcriptome‐wide association studies (TWAS). Here, we aim to integrate GWAS data on individuals of European genetic ancestry generated by the ILAE Consortium on Complex Epilepsies with gene expression data from TWAS to identify such associations for epilepsy. To achieve this, we employed MAGMA^7^ and several of its derivatives. This widely used computational tool implements a multiple linear principal components regression model to derive gene‐level *p*‐values using GWAS summary statistics as input.^7^ It is essential to distinguish gene identification in conventional GWAS from MAGMA‐based analyses. Single nucleotide polymorphism (SNP)‐level GWAS associate individual variants with disease risk, often assigning them to nearby genes without implying functional involvement. In contrast, MAGMA aggregates association signals across all SNPs within or near a gene and can integrate functional annotations such as gene expression, methylation, and chromatin interactions. This provides a more biologically informed view by highlighting genes potentially involved through regulatory or transcriptional mechanisms.

Besides MAGMA, we considered three derivatives that allow the integration multilayered data to enhance analytical power and improve the signal‐to‐noise ratio, mainly by combining multiple genetic annotations and capturing diverse mechanisms underlying genetic associations with complex traits.^8^, ^9^ We developed ME‐MAGMA, a novel adaptation that assigns risk variants to genes based on methylation quantitative trait (meQTL) data.^10^ This approach enables the exploration of epigenetic mechanisms, particularly DNA methylation, as a key player in gene regulation, influencing disease susceptibility. To incorporate tissue‐specific gene regulation, we used E‐MAGMA, an extension of MAGMA that integrates expression quantitative trait loci (eQTLs) information, mapping risk variants to putative genes based on their transcriptional activity and expression levels.^11^ This approach improves the interpretation of GWAS results by linking genetic variation to gene expression changes relevant to epilepsy and related traits. To further investigate the developmental gene regulation, we utilized Hi‐C‐coupled MAGMA (H‐MAGMA),^12^ which leverages Hi‐C chromatin interaction data to map genetic variants to their target genes.^12^

Through the integration of GWAS data, eQTL, chromatin interaction profiles, and methylation data, our multiomics analytical framework aims to identify and prioritize candidate genes related to epilepsy based on vetted hypotheses.

## 2 MATERIALS AND METHODS

### 2.1 Ethics statement

The datasets used were generated based on the following ethics statement by the ILAE Consortium on Complex Epilepsies^4^, ^5^: “We have complied with all relevant ethical regulations. All study participants provided written informed consent for the use of their data in genetic studies of epilepsy. For minors, written informed consent was obtained from their parents or legal guardians. Local institutional review boards approved study protocols at each contributing site.”

### 2.2 Data source

For this study, we used the third iteration of GWAS summary statistics, referred to as ILAE3, produced by the ILAE Consortium on Complex Genetics. These summary statistics, available online at the Epilepsy Genetic Association Database website (https://www.epigad.org/), comprise 4.9 million SNPs tested for phenotypic association using mixed model meta‐analysis in 52 538 controls and 29 944 individuals with epilepsy. In these summary statistics, epilepsy cases are classified into three major categories: FE, GGE, and unclassified epilepsy. The “all epilepsy” group comprises individuals from all three categories.^5^

Usually, the estimates based on reference data with similar ancestry to the data from which SNP *p*‐values were computed yield higher accuracy results in MAGMA. Therefore, we used the previous version of the dataset, referred to as ILAE2,^4^ as a reference panel, provided by the ILAE Consortium on Complex Epilepsies in PLINK format (Table 1). This dataset included 38 324 individuals of European ancestry, comprising 14 130 epilepsy cases and 24 194 controls. To ensure the data quality and reliability of these data, all ambiguous, nonautosomal variants and SNPs with a minor allele frequency of <.01 were excluded using PLINK 2.0.^14^, ^15^ After performing this quality control, the dataset comprised 5 535 915 variants. This dataset was used as a reference, because it is part of the ILAE3 dataset, whose summary statistics were used for the analysis.

**TABLE 1:** Data sources.

Dataset name	Sample size	Description	URL
ILAE3	Individuals: 82 482 Cases: 29 944 Controls: 52 538 SNPs GGE: 4 866 066 All: 4 880 199 Focal: 4 861 642	Third iteration of the GWAS dataset generated by ILAE Consortium^5^ Summary statistics used for MAGMA input	https://www.epigad.org/gwas_index.html
ILAE2	Individuals: 38 324 Cases: 14 130 Controls: 24 194 SNPs: 5 535 915	Second iteration of the GWAS dataset generated by ILAE Consortium on Complex Epilepsies in PLINK format^4^ and used as reference data to account for LD for MAGMA input in this study	
meQTL dataset	meQTLs: 409 372	Dataset generated by Schulz et al.^10^ Used for meQTL and CpG association information	
Illumina BeadChip dataset	cis‐CPG: 25 165 Genes: 9967	Dataset generated by Price et al.^13^ using Illumina Infinium HumanMethylation450 BeadChip array Used for CPG and gene association information in this study	https://www.ncbi.nlm.nih.gov/geo/query/acc.cgi?acc=GPL16304

Abbreviations: GGE, genetic generalized epilepsy; GWAS, genome‐wide association studies; ILAE, International League Against Epilepsy; LD, linkage disequilibrium; meQTL, methylation quantitative trait; SNP, single nucleotide polymorphism.

### 2.3 Magma

MAGMA gene analysis is performed to quantify the degree of association each gene has with the phenotype. It employs an SNP‐wise model that first analyses individual SNPs within a gene using GWAS summary statistics. It then combines the resulting SNP *p*‐values into a gene‐ or region‐level test statistic, calculated as the mean χ^2^ statistic, where a gene‐level *p*‐value is derived using a known approximation of the sampling distribution. To determine which SNP belongs to each gene, information on the gene and variant positions is needed. This information is provided by the user in the form of an annotation file. MAGMA also allows users to define a window around genes, which includes variants that are outside the gene itself but may still be relevant, such as those located within the promoter region and other regulatory areas. Additionally, MAGMA requires reference data to account for linkage disequilibrium,^7^ which in our case is the ILAE2 dataset. We used conventional MAGMA^7^ to investigate the association between common epilepsies and genetic variants located within the gene body, that is, the region of a gene that is transcribed into RNA, encompassing both exons and introns. For this, we generated an annotation file using the hg19 reference from the 1000 Genomes Project,^16^ resulting in a dataset comprising 34 434 987 SNPs mapped to 18 524 genes, to be used with MAGMA.

### 2.4 E‐magma

To explore the association between eQTL and epilepsy, we employed the E‐MAGMA framework (https://github.com/eskederks/eMAGMA‐tutorial), a derivative of the MAGMA software designed to integrate cis‐eQTL data with genetic association studies.^15^ E‐MAGMA enables the investigation of how genetic variants influence gene expression and, consequently, impact susceptibility to disease. This framework provides annotation information for 14 brain‐related tissues collected from GTEx (version 8).^11^ However, to maintain consistent analysis conditions, we focused specifically on eQTL data from hippocampal tissue. The annotation file for hippocampal tissue comprised 294 761 SNPs and 3474 genes.

### 2.5 ME‐MAGMA

meQTLs are genetic variants that influence the variation in DNA methylation patterns across the genome. To investigate the relationship between these meQTLs and genes, we developed the ME‐MAGMA (https://github.com/ashwini‐mushunuri/ME‐MAGMA) framework to identify genetically driven DNA methylation events potentially involved in epileptogenesis. As the genomic coordinates linking meQTL changes in DNA methylation at specific CpG sites were not readily available, we constructed a custom MAGMA‐compatible annotation file by integrating data from two primary sources.

The first dataset, referred to hereafter as the meQTL dataset, was an unpublished, unpruned dataset generated in one of our previous studies.^10^ It was derived from fresh‐frozen hippocampal tissue samples from 110 unrelated individuals with temporal lobe epilepsy (TLE) and comprises 984 176 cis‐meQTL SNP–CpG site pairs. Associations were identified using a false discovery rate (FDR)^17^ threshold of 5% within a cis window of ±500 kb between SNP genotypes and quantitative methylation rate (β‐value) of CpGs or 3′‐RNA expression levels, using a linear regression model implemented in Matrix eQTL.^10^

The second dataset was obtained from the Gene Expression Omnibus (GEO) under accession ID GPL16304 and belongs to the Illumina Infinium HumanMethylation450 BeadChip dataset version 1.0 (https://www.ncbi.nlm.nih.gov/geo/query/acc.cgi?acc=GPL16304).^13^ This dataset includes CpG site identifiers and their nearest associated gene symbols. To enrich this information with standardized gene identifiers and genomic coordinates, we retrieved Ensembl gene IDs, Entrez gene IDs, chromosome names, and gene start and end positions for each gene listed in the Illumina BeadChip dataset using BioMart (GRCh37 build and Ensembl Genes version 114).^16^

This query yielded 11 092 Ensembl gene IDs corresponding to 9967 unique gene symbols. These Ensembl IDs were mapped to their respective CpG site IDs in the Illumina BeadChip dataset using R v4.3.2 and tidyverse v2.0.^18^, ^19^ With the CpG IDs as a common key, we subsequently linked the Ensembl gene information to the meQTL SNPs from the meQTL dataset, thereby establishing associations between cis‐meQTLs and genes via their shared CpG sites.

This integrative mapping resulted in a dataset comprising 25 165 unique cis‐CpG sites and 409 372 associated meQTL SNPs, leading to a total of 1 350 219 SNP–CpG–gene associations. The final dataset was formatted into a MAGMA‐compliant annotation file for downstream gene‐based analysis, allowing for a precise investigation of meQTL–gene relationships.

### 2.6 H‐MAGMA

To capture the dynamic changes in chromatin interactions that occur during brain development and to gain insights into the genetic factors contributing to epilepsy, we utilized H‐MAGMA.^12^ The H‐MAGMA annotation files, which comprise chromatin interaction data, were created by assigning exonic and promoter SNPs to their corresponding target genes based on their genomic locations, using the Gencode v26 (GRCh37) gene model.^12^ This gene model encompasses a total of 58 219 genes, which include protein‐coding genes, small noncoding RNA genes, and various types of pseudogenes (including processed, unprocessed, unitary, and polymorphic pseudogenes).

The annotation information is available on GitHub (https://github.com/thewonlab/H‐MAGMA) for two developmental stages—adult and fetal brain—as well as for three cell types: induced pluripotent stem cell (iPSC)‐derived neurons, iPSC‐derived astrocytes, and cortical neurons. However, in this study, we only utilized datasets from adult and fetal brain tissues, referred to as H‐MAGMA Adult and H‐MAGMA Fetal, respectively. The annotation information for adult brain tissue contains 3 943 744 SNPs and 54 341 genes and pseudogenes, and the fetal brain tissue annotation includes 3 978 004 SNPs and 54 332 genes and pseudogenes.

### 2.7 Gene‐level analysis

We conducted a comprehensive gene‐level analysis using various regulatory genomic features described above to identify candidate genes that are associated with epilepsy. To ensure consistency in the computational parameters, we utilized MAGMA's “snp‐wise = mean” mode for all methods. This approach averages the effects of all SNPs associated with a gene, providing a more balanced and representative measure of their collective influence.

**FIGURE 1:**
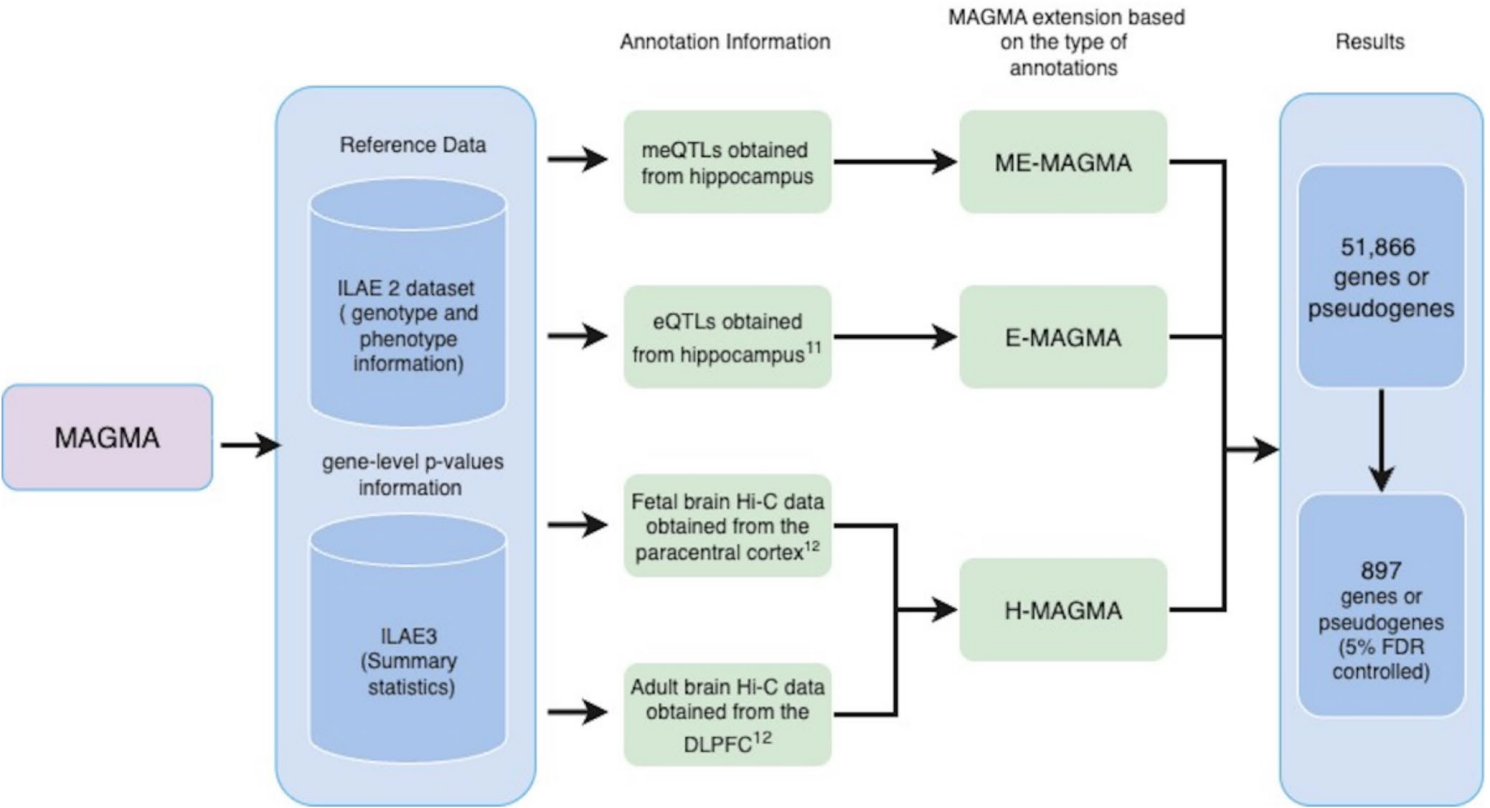
Flowchart describing various dataset usage at different stages of methodology. DLPFC, dorsolateral prefrontal cortex; eQTL, expression quantitative trait locus; FDR, false discovery rate; ILAE, International League Against Epilepsy; meQTL, methylation quantitative trait.

The resulting gene‐level associations were FDR‐adjusted and filtered using an FDR threshold of ≤.05 to ensure statistical robustness and minimize the risk of false positives.^17^ This analysis was conducted separately using the ILAE3 summary statistics for focal epilepsy, genetic generalized epilepsy, and “all epilepsy” (Figure 1).

### 2.8 Gene prioritization

To identify key candidates within this extensive list, we used VarElect,^20^ which is a phenotype‐based variation prioritization tool. It prioritizes genes based on their potential association with specific phenotypes, using factors such as pathway interactions and gene expression patterns. This approach enabled us to identify several critical genes as promising candidates (Table 2) for further investigation. The keyword “epilepsy” was used to associate the phenotype with the genes.

**TABLE 2:** Number of associated genes and pseudogenes following 5% Bonferroni and FDR correction.

Method	FDR‐corrected value			Bonferroni‐corrected value		
	GGE	FE	All	GGE	FE	All
ME‐MAGMA	101	1	9	16		1
E‐MAGMA	51	1	16	14	1	1
MAGMA	236	2	29	35	1	6
H‐MAGMA adult	534	0	38	60		8
H‐MAGMA fetal	489	0	47	59		7

Abbreviations: FDR, false discovery rate; FE, focal epilepsy; GGE, genetic generalized epilepsy.

## 3 RESULTS

### 3.1 Genetic generalized epilepsies

In this gene‐level association study using MAGMA and its extensions, we analyzed a total of 51 866 genes and pseudogenes. Pseudogenes are genomic DNA sequences resembling mutated or truncated versions of functional genes. Emerging evidence suggests that many pseudogenes are transcribed at nonrandom levels, with some transcripts exhibiting biological functions, warranting their classification as a subclass of functional long noncoding RNAs.^21^

**FIGURE 2:**
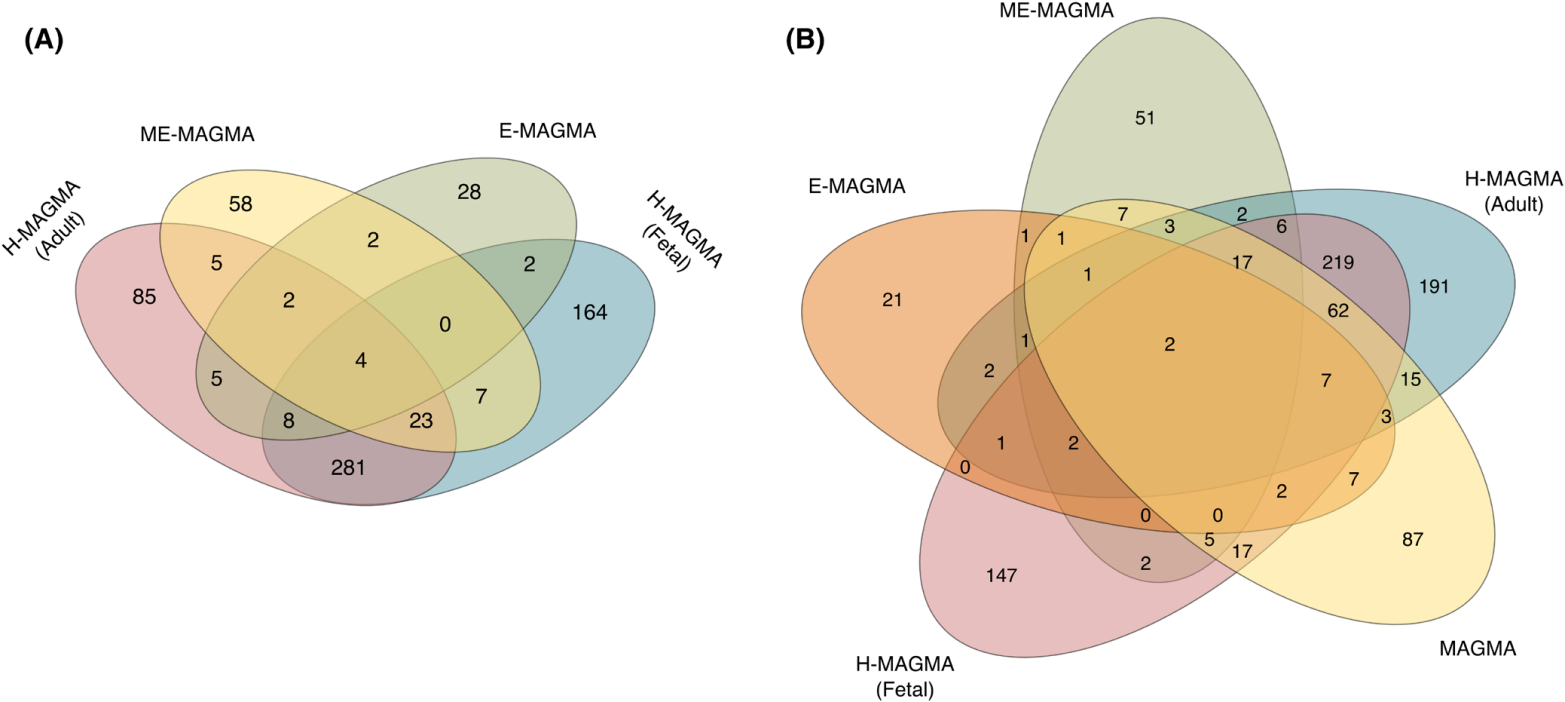
(A) Venn diagram illustrating the intersection of results obtained from H‐MAGMA, E‐MAGMA, and ME‐MAGMA, highlighting both shared and unique findings. (B) Venn diagram showing the overlap between the results from MAGMA and its extensions. The overlap includes genes associated with genetic generalized epilepsy at the 5% false discovery rate level for ME‐MAGMA (*n* = 58), E‐MAGMA (*n* = 28), H‐MAGMA Adult (*n* = 85), and H‐MAGMA Fetal (*n* = 164), respectively. Only four genes—*DPM2, VAMP2, MPI*, and *ARRDC1*—were common across four analyses, and only *DPM2 and VAMP2* were common across all five analyses, with *VAMP2* being the only one previously reported in the original study.

After applying a 5% FDR correction, we identified 897 genes associated with GGE, as shown in Table 2. Notably, 18 of these genes had previously been implicated in SNP‐level GWAS conducted by the ILAE Consortium on Complex Genetics, indicating a strong overlap between our findings and established genetic associations. The overlapping genes include *AP3D1, ARHGEF15, ATXN1, CACNA2D2, CDK5RAP3, FANCL, GABRA2, KCNIP2, KCNN2, PCDH7, RBFOX1, RMI1, SCN1A, SCN2A, SPOCK1, STX1B, TMPRSS15*, and *PNPO*. Furthermore, some overlap was observed across all calculations, as illustrated in Figure 2.

In the H‐MAGMA Adult and H‐MAGMA Fetal dataset analyses, we identified 413 and 489 genes and pseudogenes, respectively, 316 of which are common to both studies (see Supplemental Data 1). Among our candidate genes controlled for 5% FDR in both H‐MAGMA and MAGMA analyses, we found *GNAI2, GNAT1, LSMEM2, RASSF1, SEMA3F, SLC38A3*, and *IFRD2*, which are located at locus 3p21.31 (see Supplemental Data 1).

**FIGURE 3:**
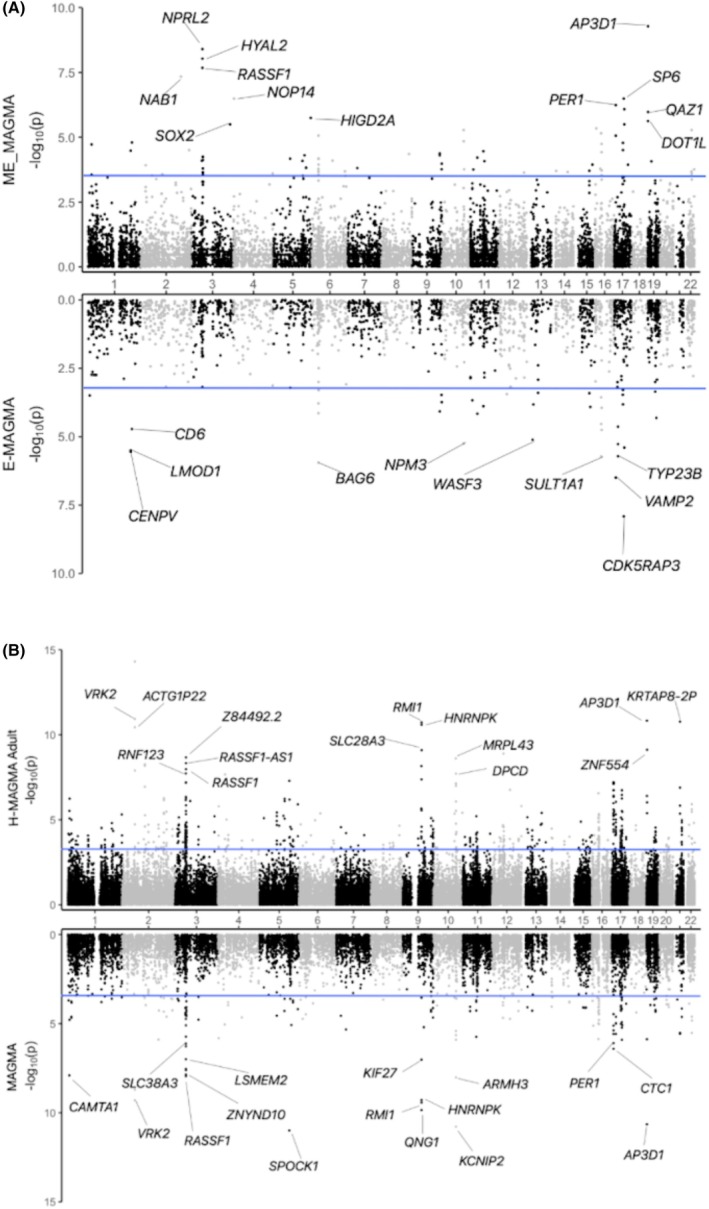
Miami plots illustrating *p*‐values from gene‐based analyses. In both panels, the blue lines mark the threshold corresponding to a 5% false discovery rate (FDR), and genes with the lowest FDR‐corrected *p*‐values are labeled. (A) ME‐MAGMA and E‐MAGMA analyses in hippocampal tissue (nominal *p*‐value thresholds: 5.26 × 10^−4^ and 9.98 × 10^−4^, respectively), with most top candidate genes clustered on chromosomes 3 and 17. (B) H‐MAGMA Adult brain tissue and MAGMA analyses (nominal *p*‐value thresholds: 5.75 × 10^−4^ and 7.19 × 10^−4^, respectively), showing a notable clustering of top candidate genes on chromosome 3, suggesting a higher mutation rate that may contribute to epilepsy.

In the ME‐MAGMA analysis, we identified *AP3D1, HYAL2, NPRL2, RASSF1*, and *NAB1* as the candidates with the lowest *p*‐values (see Supplemental Data 1). Among these, *NPRL2*, *HYAL2*, and *RASSF1* are located at locus 3p21.31 (Figure 3A), suggesting the potential influence of meQTLs in linking this locus to GGE.

Additionally, in Figure 3A, genes appear clustered on chromosome 17, suggesting a potential methylation‐related effect specific to this chromosome. This is further supported by the identification of several genes, such as *PER1* (17p13.1), *SP6* (17q21.32), and *MYCBPAP* (17q21.33), in the ME‐MAGMA analysis. Similarly, the E‐MAGMA analysis revealed additional candidate genes on chromosome 17, including *TVP23B*, *TRIM16L*, and *PRPSAP2* at 17p11.2. We also identified genes common to both E‐MAGMA and H‐MAGMA on chromosome 17, such as *CDK5RAP3* at 17q21.32, *VAMP2* at 17p13.1, and *SPATA20* at 17q21.33.

### 3.2 Focal epilepsies

For FEs, our analysis suggests three genes that had not been previously reported in the ILAE Consortium studies.^4^, ^5^ These genes include *FANCD2P2* and *CUL4A*, identified through MAGMA, and *PROZ*, identified through ME‐MAGMA and E‐MAGMA (Supplemental Data 1).

### 3.3 All epilepsies

Using the “all epilepsy” summary statistics, we observed associations with 97 genes (Supplemental Data 1). Among these, five genes had already been recognized in the original study.^4^, ^5^ These genes include *KCNIP2, RMI1, SCN1A*, and *FANCL*, identified through H‐MAGMA Adult, H‐MAGMA Fetal, and MAGMA, as well as *SPOCK1*, identified through H‐MAGMA Adult, H‐MAGMA Fetal, MAGMA, and ME‐MAGMA. Additionally, *ARHGEF15*, identified through H‐MAGMA Fetal and MAGMA, was also observed in our analysis of GGE.

## 4 DISCUSSION

The etiology of epilepsy is complex, often involving multiple genetic variations that disrupt neural pathways and contribute to seizure susceptibility. Focusing on a single gene or pathway is not always sufficient to fully capture the genetic underpinnings of epilepsy. Accordingly, a comprehensive approach that considers the cumulative effects of multiple genetic variants is essential for understanding its genetic architecture.^5^, ^22^ In this study, we present evidence‐based hypotheses regarding the contribution of these variants to epilepsy risk. To investigate their impact, we used the MAGMA statistical framework and its derivatives, E‐MAGMA, H‐MAGMA, and ME‐MAGMA, which integrate diverse datasets such as transcriptional profiles, chromatin interaction maps, and epigenetic marks, respectively, to identify genes influenced by these genetic variations. We analyzed chromatin interaction maps from both adult and fetal brains using H‐MAGMA, following the findings of Sey et al.,^12^ which show that psychiatric and neurological disorders tend to have greater heritability enrichment in the fetal brain than in the adult brain, especially for childhood onset disorders like attention‐deficit/hyperactivity disorder and autism spectrum disorder, highlighting their neurodevelopmental origins.^12^ A similar integrative gene‐level approach, using multiple variations of MAGMA, has been applied in a study of traumatic brain injury,^23^ which supports the validity of our multiomic approach to uncovering regulatory mechanisms underlying neurological disorders.

ME‐MAGMA is a novel method for identifying epilepsy‐associated meQTL SNPs, offering insights into the epigenetic mechanisms contributing to seizure susceptibility and supporting the development of targeted therapies using data from hippocampal tissues obtained from TLE patients. Using these data from annotation files in MAGMA is acceptable because these annotations are not used to determine case–control status. Instead, they help highlight genomic regions that are biologically active or relevant in the context of epilepsy.

When analyzing GWAS data for GGE, incorporating epigenomic marks derived from TLE patients can reveal whether GGE‐associated genetic variants are enriched in regions of the genome that are active in epileptic brain tissue. This approach can provide valuable insights into the regulatory mechanisms that may be shared between TLE and GGE, or it may highlight distinct pathways involved in each epilepsy subtype. One gene of interest is *NPRL2*, which has already been associated with FE^24^ but has not yet been linked to GGE. This suggests shared or distinct regulatory mechanisms of the same gene based on variations between the two types of epilepsy.

The value of integrating methylation data in epilepsy research is further supported by a study from Kobow et al.,^25^ which used DNA methylomes and transcriptomes from focal cortical dysplasia (FCD) specimens and controls to distinguish major FCD subtypes from TLE and nonepileptic cases.^25^ These findings highlight the relevance of methylation in disease classification and mechanisms. Together, ME‐MAGMA and similar epigenomic strategies underscore the potential of methylation‐based analyses to deepen our understanding of different types of epilepsies.

### 4.1 Multiple testing correction

Both the FDR and the Bonferroni correction are methods used to adjust for multiple testing when interpreting MAGMA results. FDR controls for the expected proportion of false positives among the significant results and is used to interpret gene sets. Bonferroni controls the probability of even one false positive. FDR is therefore the preferred method for our exploratory analysis, particularly for gene set overlap comparisons between different MAGMA derivatives (Figure 2). However, we have considered both correction methods in Table 2 in summary and Supplemental Data 1 in detail.

### 4.2 Gene‐based associations for genetic generalized epilepsies

#### 4.2.1 Voltage‐gated calcium channels

Our MAGMA analysis found voltage‐gated calcium channel (VGCC) genes, which are widely expressed in the central nervous system and regulate neuronal excitability and intracellular Ca^2+^ dynamics, to be associated with GGE. Calcium ions mediate key cellular processes such as excitation–transcription, excitation–contraction, and excitation–exocytosis coupling, especially in synaptic transmission.^26^ We found association between GGE and *CACNA2D2* and *CACNB2*, which encode the α2δ2 and β2 VGCC subunits. *CACNA2D2* has been previously linked to GGE,^5^ and we reaffirm this connection in the current study. Additionally, *CACNA2D2* has been associated with monogenic forms of developmental and epileptic encephalopathy.^27^, ^28^ On the other hand, *CACNB2* has not been associated with GGE specifically yet, but its upregulation is linked to TLE,^29^ suggesting that calcium channel subunit dysfunction may contribute broadly to various epilepsy subtypes.

#### 4.2.2 Voltage‐gated potassium channels

Potassium channel activation plays a crucial role in repolarizing the cell membrane after excitation. Among the genes coding for voltage‐gated potassium channels identified in this study, *KCND3, KCNJ4*, and *KCNT1* were newly found to be associated with GGE, whereas *KCNIP2* and *KCNN2* have been described as potential candidates in prior ILAE consortium studies.^4^, ^5^ *KCNT1*, identified through H‐MAGMA Adult, encodes the sodium‐activated potassium channel subunit K_Na_1.1. Gain‐of‐function variants in *KCNT1* are linked to early onset epileptic encephalopathies, including early infantile developmental and epileptic encephalopathy (DEE), infantile epileptic spams syndrome, epilepsy of infancy with migrating focal seizures, and sleep‐related hypermotor epilepsy.^30^, ^31^ *KCND3*, identified via MAGMA analysis, is associated with Dravet‐like syndrome, DEE, intellectual disability, and cardiac arrhythmias.^32^, ^33^ *KCNJ4*, consistently identified across our GGE analyses, encodes the inwardly rectifying potassium channel subunit Kir2.3, which has been implicated in kainate‐induced TLE through its upregulation.^34^ Even though the monogenic syndromes associated with these genes are quite diverse, identification of these genes in our study suggests that common variants may contribute to the phenotype of GGE.

#### 4.2.3 Transmembrane protein‐coding genes

Our GGE analysis identified several transmembrane protein‐coding genes across different analytical frameworks. Notably, *TMEM107* was detected using both H‐MAGMA and MAGMA, whereas *TMEM186* was identified in H‐MAGMA Fetal analyses. *TMEM115* and *TMPRSS15* emerged from MAGMA, and *TMEM177* was highlighted by E‐MAGMA. Notably, *TMPRSS15* has previously been linked to GGE in an ILAE study.^5^

Mutations in *TMEM107* have been associated with ciliopathies such as Meckel syndrome and Joubert syndrome, conditions often accompanied by neurological abnormalities, including developmental delays and brain malformations.^35^ Investigating this gene could provide novel insights into the role of chromatin interactions and their developmental effects on neurological pathways. *TMEM177* mutations are implicated in mitochondrial dysfunction, a key factor in various epilepsy subtypes, including infantile spasms, myoclonic epilepsy with ragged‐red fibers, and TLE.^36^, ^37^ Although *TMEM115* and *TMEM186* have not been directly linked to GGE in previous publications, the positioning of *TMEM115* at the 3p21.31 locus, known to harbor multiple epilepsy‐related genes, suggests its potential relevance as a candidate gene for epilepsy.

#### 4.2.4 Zinc finger proteins

Zinc finger proteins (ZNFs) are involved in various cellular processes, including transcriptional regulation and DNA repair.^38^ In this study, *ZBTB32*, *ZNF668*, and *ZNF629* were identified through E‐MAGMA, H‐MAGMA (Adult), and ME‐MAGMA analyses, respectively.

*ZBTB32* is linked to DEE 52,^39^, ^40^ and deletions at its locus are associated with generalized epilepsy with febrile seizures plus (GEFS+) type 1. Mutations due to eQTLs in *ZBTB32* can disrupt gene regulation, potentially contributing to GGE.

*ZNF668* is crucial for DNA damage repair, with its deficiency leading to severe developmental issues and being linked to GEFS+ type 9.^24^, ^41^ *ZNF629* encodes for a transcription factor regulating RNA polymerase II, with variants associated with intellectual developmental disorders and^42^ juvenile myoclonic epilepsy.^40^ Epigenetic mutations in such factors can alter gene expression and contribute to seizure disorders.

These findings suggest a role for epigenetic and transcriptional dysregulation in the etiology of GGE and its subtypes.

#### 4.2.5 Other noteworthy genes

Using the ME‐MAGMA approach, we found other genes such as *NPRL2* and *SOX2*, that play crucial roles in the molecular pathways underlying seizure susceptibility and brain development. Loss‐of‐function mutations in *NPRL2* disrupt the GATOR1 complex, leading to hyperactivation of the mTORC1 pathway, a significant contributor to seizure development.^43^ Additionally, this gene interacts with epigenetic regulators essential for chromatin modification and gene expression, underscoring the intricate relationship between signaling pathways and epigenetic mechanisms in neuronal function.^44^

SRY‐related HMG‐box 2 (SOX2) is a transcription factor vital for hippocampal development, regulating neural stem cell proliferation. Epigenetic dysregulation of *SOX2* is linked to neurological disorders caused by impaired hippocampal development and synaptic plasticity, potentially contributing to epileptogenesis.^45^, ^46^

We also identified additional genes, such as *HYAL2*, *RASSF1*, and *NAB1*, that play significant roles in brain function.^47^, ^48^, ^49^ Although *RASSF1* is not directly associated with GGE yet, its epigenetic signatures are linked to other epilepsy‐causing genes such as *PTEN*, *BRAF*, *HRAS*, and *KRAS*, which have already been identified.^50^, ^51^ However, the relationship of *HYAL2* and *NAB1* with epilepsy remains poorly understood, warranting further investigation to elucidate their potential involvement in neural function.

*EHMT1* encodes a histone methyltransferase that methylates H3K9. *EHMT1*‐associated chromatin modification has been linked to neurodevelopmental disorders and intellectual disability.^41^, ^52^

Similarly, *UBTF*, a gene identified through all our analyses except E‐MAGMA, has been directly associated with a severe neurodevelopmental disorder known as *UBTF* neuroregression syndrome, also referred to as childhood onset neurodegeneration with brain atrophy. Mutations in *UBTF* disrupt ribosome biogenesis, impairing protein synthesis and cellular function, which contributes to the development of neurodegenerative symptoms, including seizures.^53^ These findings position *UBTF* as a potential candidate for epilepsy‐related studies and highlight the possible role of eQTL in the disorder's pathogenesis.

#### 4.2.6 Loci 3p21.31 and 17q21.31–32

In this study, we identified multiple candidate genes located at the loci 3p21.31 and 17q21.31–32. Variations in these genes within the same region can lead to polygenic or oligogenic effects, meaning that although each gene may contribute just a small effect, collectively they can surpass a threshold that influences traits like epilepsy. Additionally, the genes that are situated close to one another may share enhancers or promoters, which is often found in coexpressed genes, allowing a single variant to impact the expression of multiple genes.

Deletions in the 3p21.31 locus have been associated with neurodevelopmental disorders, such as intellectual disability, autism spectrum features, and epilepsy.^54^ Moreover, the *CACNA2D2* gene, which encodes a subunit of voltage‐dependent calcium channels, has also been implicated in epilepsy through GWAS.^5^

Likewise, deletions in the 17q21.31 locus are linked to a genetic disorder called Koolen–de Vries syndrome.^55^ The nearby 17q21.32 locus contains the *PNPO* gene, where recessive mutations can lead to severe pyridoxal phosphate deficiency, resulting in neonatal or infantile seizures, often showing as status epilepticus during fevers. This region also includes genes such as *CBX1* and *CDK5RAP3*, which are involved in neuronal differentiation and migration, correlating with cortical microdysgenesis observed in juvenile myoclonic epilepsy and other forms of GGE.^56^ Additionally, locus 17q21.32 has been reported as a potential pleiotropic locus for FE and GGE.^57^

Identification of multiple genes at these loci (Figure 3A) highlights the potential role of meQTLs and eQTL in modulating the relationship between the chromosomal regions 3p21.31 and 17q21.31–32 and GGE. Furthermore, the clustering of genes on chromosome 3, as depicted in the Miami plot from the H‐MAGMA analysis (Figure 3B), suggests that these regions may influence neurodevelopmental processes that contribute to susceptibility to GGE.

### 4.3 Gene‐based associations for all epilepsies

In addition to the genes identified for GGE, our analysis on the “all epilepsy” dataset identified genes like *HNRNPK*, *MAGI2*, *PACS1*, and *TTC21B* using MAGMA, E‐MAGMA, and ME‐MAGMA, respectively.

*HNRNPK* gene encodes heterogeneous nuclear ribonucleoprotein K, a multifunctional protein involved in various aspects of RNA metabolism. Mutations or haploinsufficiency in *HNRNPK* have been linked to a rare autosomal dominant neurodevelopmental disorder known as Au–Kline syndrome and autism spectrum disorder.^58^, ^59^

*MAGI2* (membrane associated guanylate kinase, WW and PDZ domain containing 2) has been implicated in certain forms of epilepsy, particularly infantile epileptic spasms syndrome, a severe epilepsy syndrome occurring in the first year of life. Research has identified deletions in the *MAGI2* gene on chromosome 7q11.23‐q21.1 in patients with infantile epileptic spasms syndrome, suggesting a potential role in the disorder's pathogenesis.^60^

Mutations in the *PACS1* gene are associated with Schuurs–Hoeijmakers syndrome (PACS1 syndrome), a rare neurodevelopmental disorder characterized by intellectual disability, developmental delays, distinctive facial features, and seizures.^61^ The identification of *PACS1* through E‐MAGMA suggests that epilepsy risk may be modulated by eQTLs influencing gene regulation.

Studies show that the cis‐regulated expression level of *TTC21B* is associated with epilepsy, indicating that risk variants may confer epilepsy risk by regulating the expression of this gene.^6^ The identification of *TTC21B* in ME‐MAGMA highlights the involvement of DNA methylation mechanisms.

The identification of pseudogenes *ACTG1P22* and *KRTAP8‐2P* in our H‐MAGMA Adult and Fetal analysis suggests that these genes may provide valuable insights into the hereditary and developmental mechanisms underlying epilepsy.^62^

### 4.4 Gene‐based associations for focal epilepsies

In our analysis of a focal epilepsy dataset, we identified *FANCD2P2*, *CUL4A*, and *PROZ* as genes and pseudogenes of interest. *FANCD2P2* is related to *FANCD2*, a key component of the Fanconi anemia pathway involved in DNA repair processes.

The *CUL4A* gene encodes a critical component of the cullin‐RING ubiquitin ligase complex, which regulates protein degradation and cell cycle control. The *PROZ* gene encodes protein Z, a glycoprotein that plays a regulatory role in blood coagulation.

Although these genes are recognized for their essential functions in the human brain, mutation or dysregulation in these loci has not yet been directly linked to neurological disorders, including epilepsy. The identification of these genes in our study may serve as a foundation for future research into their potential roles in the mechanisms underlying neurological diseases. Moreover, incorporating disease‐specific methylation data can enhance the discovery of more relevant genes associated with focal epilepsies.^25^

## 5 CONCLUSIONS

In this study, we aimed to uncover new genes associated with epilepsy by integrating genetic variants through multiple layers of gene regulations including positional, transcriptional, and epigenetic. Rather than focusing solely on overlapping results across methods, we emphasized the unique regulatory insights each approach provides, as these can offer critical clues and directions for future research. To achieve this, we utilized a range of MAGMA‐based methodologies, including MAGMA, E‐MAGMA, H‐MAGMA, and a novel approach developed in this study, ME‐MAGMA. These methods allowed us to systematically analyze the genetic architecture of epilepsy, particularly GGE, by incorporating genomic, transcriptomic, and epigenomic information. Standard MAGMA provided gene‐level associations from GWAS data. E‐MAGMA and H‐MAGMA extended this by integrating gene expression and chromatin interaction information, respectively, whereas ME‐MAGMA introduced a new layer of analysis by incorporating meQTL data to capture epigenetic regulation. Together, these complementary approaches revealed candidate genes involved in seizure susceptibility, highlighting the complex interplay between genetic variants and different regulatory mechanisms (Table 3).

**TABLE 3:** List of key candidate genes selected based on their FDR, available literature, and VarElect score.

	Genes	VarElect score^a^	Disease‐causing likelihood^b^	ME‐MAGMA	E‐MAGMA	H‐MAGMA adult	H‐MAGMA fetal	MAGMA
GGE	*CACNB2*	5.25	47.24					4.02 × 10^−6^
	*ZNF629*	3.80	65.59	1.17 × 10^−2^				
	*ZNF668*	1.61	66.64			1.64 × 10^−2^		3.097 × 10^−2^
	*ZBTB32*	1.39	54.25		2.57 × 10^−2^			
	*KCND3*	18.70	88.13					1.07 × 10^−2^
	*KCNT1*	105.48	77.33			3.82 × 10^−2^		
	*TMEM107*	1.16	72.83			2.54 × 10^−3^	2.79 × 10^−3^	3.42 × 10^−2^
	*TMEM186*	1.18	46.82			3.02 × 10^−2^	3.25 × 10^−2^	
	*TMEM115*	1.43	73.97					1.44 × 10^−2^
	*NPRL2*	36.27	87.30	1.98 × 10^−5^				
	*SOX2*	15.94	70.26	2.25 × 10^−3^				
	*RASSF1*	2.12	26.16	5.21 × 10^−5^		2.07 × 10^−5^	2.62 × 10^−5^	1.45 × 10^−2^
	*NAB1*	3.68	81.39	9.08 × 10^−5^		6.35 × 10^−4^	1.98 × 10^−4^	2.05 × 10^−3^
	*UBTF*	13.95	91.65	1.07 × 10^−2^		3.40 × 10^−3^	1.42× 10^−3^	5.94 × 10^−3^
	*EHMT1*	17.88	68.20					3.12× 10^−2^
All	*HNRNPK*	3.33	91.33			3.40 × 10^−3^		3.00 × 10^−3^
	*MAGI2*	15.56	80.25					1.30 × 10^−2^
	*PACS1*	16.22	84.75		4.44 × 10^−2^			
	*TTC21B*	18.35	29.84	4.05 × 10^−2^				
Focal	*PROZ*	.11	42.49	5.75 × 10^−2^	1.44 × 10^−2^			
	*CUL4A*	.11	53.06					2.72 × 10^−2^

Abbreviation: GGE, genetic generalized epilepsy.

^a^ VarElect score is an indication of the strength of the connection between the gene and the queried phenotype(s). The main purpose of the score is to enable ranking and prioritizing the list of queried genes by relevance to the queried phenotype(s).^20^

^b^ Disease‐causing likelihood reflects the principle that a variant in a gene with high mutation intolerance is more likely to be disease‐causing. Detailed description can be found in the referenced publication.^20^

Here, we identified both known and novel genes associated with epilepsy. For each gene, we provide information regarding the specific types of genetic variation that may lead to the observed trait. Gaining deeper insights into these variations, and whether they impact on epigenetic mechanisms or developmental processes, can offer more targeted and biologically meaningful interpretations. For example, we observed overlapping genetic variants within several of these genes when mapped using both the H‐MAGMA Adult and Fetal brain‐specific annotation frameworks. This convergence suggests that these genes may exert regulatory or functional effects across multiple stages of neurodevelopment, potentially influencing susceptibility to epilepsy at different stages of life.

Our findings also highlight the critical role of ion channel dysfunction and transcriptional dysregulation in the development of epilepsy. We identified several key genes, such as *KCNT1, CACNB2, NPRL2*, and *SOX2*, that comprise common variants associated with epilepsy. In total, we uncovered a set of 879 genes and pseudogenes impacted by diverse genomic mechanisms, broadening the pool of potential therapeutic targets.

This research reinforces the critical role of epigenetic and transcriptional regulation in epilepsy, underscoring the necessity for multiomic approaches to unravel its complex pathogenesis. Although this study offers a set of robust, data‐driven hypotheses, it also underscores the need for further functional validation and mechanistic exploration. Continued investigation into these genes, particularly in the context of different epilepsy subtypes and neurodevelopmental timelines, will be crucial for deepening our understanding of their roles in the epileptogenesis process.

## AUTHOR CONTRIBUTIONS

**Ashwini Mushunuri**: Writing—original draft preparation; statistical analysis; methodology; visualization. **Oluyomi Adesoji**: Review of the original draft. **Roland Krause**: Data curation; review of the original draft. **Patrick May**: Review of the original draft. **Holger Lerche**: Review of the original draft. **Albert Becker**: Resources; review of the original draft. **Daniela Grimm**: Review of the original draft. **International League Against Epilepsy Consortium on Complex Epilepsies**: Resources; review of the original draft. **Michael Nothnagel**: Supervision; writing—review & editing. **Herbert Schulz**: Conceptualization; supervision; funding acquisition; methodology; writing—review & editing.

## FUNDING INFORMATION

This research was funded by the German Research Foundation and the Luxembourg Fond Nationale de Recherche as a part of the research unit FOR‐2715 (grants SHU3585/1.1, NO755/6‐1, NO755/13‐1, Le1030/16‐2, INTER/DFG/21/16394868 MechEPI2, BE 2078/5‐1, BE 2078/10‐2).

## CONFLICT OF INTEREST STATEMENT

The authors report no conflict of interest. We confirm that we have read the Journal's position on issues involved in ethical publication and affirm that this report is consistent with those guidelines.

## Supporting information


Appendix S1.

Data S1.

## Data Availability

Research data are not shared.
