# Supplementary material for: Genetic risk factor identification for common epilepsies guided by integrative omics data analysis
Source: Epilepsia. 2025 Nov 30;67(3):1406–20. doi: 10.1111/epi.70021 (PMC13007835; doi:10.1111/epi.70021)
Supplement: Supplementary file 1 — Appendix S1. [file EPI-67-1406-s001.docx]

**The International League Against Epilepsy Consortium on Complex Epilepsies**

Bassel Abou-Khalil^1^, Oluyomi M Adesoji^2^, Zaid Afawi^3^, Elisabetta Amadori^4, 5^, Alison Anderson^6, 7^, Danielle M Andrade^8^, Grazia Annesi^9^, Pauls Auce^10^, Andreja Avbersek^11^, Melanie Bahlo^12-14^, Mark D Baker^15^, Ganna Balagura^4, 5^, Simona Balestrini^11, 16-18^, Carmen Barba^17, 18^, Karen Barboza^19^, Fabrice Bartolomei^20^, Thomas Bast^21, 22^, Larry Baum^23^, Tobias Baumgartner^24^, Betül Baykan^25^, Nerses Bebek^26, 27^, Albert J Becker^28^, Felicitas Becker^29^, Caitlin A Bennett^30^, Samuel F Berkovic^30, 31^, Ahmad Beydoun^32^, Francesca Bisulli^33, 34^, Ingo Borggraefe^35, 36^, Christian Bosselmann^37^, Jonathan P Bradfield^38, 39^, Lawrence C Brody^40^, Russell J Buono^38, 41, 42^, Robyn M Busch^43-45^, Ciarán Campbell^46, 47^, Ellen Campbell^48^, Laura Canafoglia^49^, Barbara Castellotti^50^, Claudia B Catarino^11^, Gianpiero L Cavalleri^46, 47^, Felecia Cerrato^51^, Francine Chassoux^52^, Siwei Chen^53, 54^, Stacey S Cherny^23, 55^, Ching-Lung Cheung^56^, Krishna Chinthapalli^11^, I-Jun Chou^57^, Seo-Kyung Chung^58, 59^, Andrew J Cole^60^, Antonietta Coppola^61^, Mahgenn Cosico^62, 63^, Patrick Cossette^64^, John J Craig^65^, Norman Delanty^46, 47, 66^, Chantal Depondt^67^, Orrin Devinsky^68^, Lidia Di Vito^33^, Dennis J Dlugos^62^, Viola Doccini^17^, Colin P Doherty^69-71^, Hany El-Naggar^46, 47, 66^, Christian E Elger^72^, Colin A Ellis^73^, Johan G Eriksson^74^, Annika Faucon^75^, Yen-Chen A Feng^76^, Lisa Ferguson^44^, Thomas N Ferraro^41, 77^, Martha Feucht^78^, Beata Fonferko-Shadrach^15^, Francesco Fortunato^79^, Jacqueline A French^80^, Elena Freri^81^, Monica Gagliardi^82^, Antonio Gambardella^79^, Tania Giangregorio^83^, Tracy Glauser^84^, Alicia Goldman^85^, Tiziana Granata^81^, Renzo Guerrini^17, 18, 86^, Namrata Gupta^54^, Kevin F Haas^1^, Hakon Hakonarson^38, 87^, Kerstin Hallmann^24, 88^, Emadeldin Hassanin^89, 90^, Manu Hegde^91^, Erin L Heinzen^92, 93^, Ingo Helbig^62, 63, 73, 94-96^, Christian Hengsbach^37^, Kjell Heuser^97^, Shinichi Hirose^98^, Edouard Hirsch^99^, Daniel P Howrigan^51, 53, 54^, Michele Iacomino^5^, Lukas L Imbach^100^, Yushi Inoue^101^, Jennifer Jamnadas-Khoda^11, 102^, Lara Jehi^44, 103^, Michael R Johnson^104^, Reetta Kälviäinen^105, 106^, Yoichiro Kamatani^107^, Moien Kanaan^108^, Masahiro Kanai^53, 54, 109^, Bülent Kara^110^, Symon M Kariuki^111-113^, Dorothee Kasteleijn-Nolst Trenite^114^, Mitsuhiro Kato^115^, Josua Kegele^37^, Nathalie Khoueiry-Zgheib^116^, Chontelle King^117^, Heidi E Kirsch^91^, Karl M Klein^118-121^, Gerhard Kluger^122, 123^, Robert C Knowlton^91^, Bobby P C Koeleman^114^, Amos D Korczyn^3^, Andreas Koupparis^124^, Ioanna Kousiappa^124^, Roland Krause^89^, Martin Krenn^125^, Heinz Krestel^126^, Ilona Krey^127^, Wolfram S Kunz^24, 128^, Gerhard Kurlemann^129^, Ruben Kuzniecky^130^, Patrick Kwan^6, 7, 131^, Angelo Labate^132^, Austin Lacey^46, 47, 66^, Dennis Lal^43, 44, 51^, Zied Landoulsi^89, 133^, Yu-Lung Lau^134^, Stephan Lauxmann^37, 135^, Stephanie L Leech^30^, Johannes R Lemke^127^, Holger Lerche^37^, Gaetan Lesca^136^, Costin Leu^11, 43, 51^, David Lewis-Smith^47, 137^, Gloria H-Y Li^56, 138^, Qingqin S Li^139^, Laura Licchetta^33^, Kuang-Lin Lin^57^, Dick Lindhout^114, 140^, Iscia Lopes-Cendes^141^, Daniel H Lowenstein^91^, Colin H T Lui^142^, Francesca Madia^5^, Anthony G Marson^143^, Patrick May^89^, Christopher M McGraw^144^, Davide Mei^17, 18, 86^, James L Mills^145^, Raffaella Minardi^33^, Rikke S Møller^146, 147^, Anne M Molloy^70^, Martino Montomoli^17, 86^, Barbara Mostacci^33^, Lorenzo Muccioli^33, 34^, Hiltrud Muhle^95^, Karen Müller-Schlüter^148^, Imad M Najm^44, 45^, Wassim Nasreddine^32^, Bernd Neubauer^149^, Charles RJC Newton^111-113^, Michael Nothnagel^2, 150^, Peter Nürnberg^2^, Terence J O’Brien^6, 7^, Yukinori Okada^109, 151^, Karen L Oliver^12, 13, 30^, Çiğdem Özkara^152^, Faith Pangilinan^40^, Savvas S Papacostas^124^, Elena Parrini^17, 86^, Manuela Pendziwiat^94, 95^, William O Pickrell^15, 153^, Tommaso Pippucci^154^, Annapurna Poduri^155^, Rob H W Powell^15, 153^, Michael Privitera^156^, Rodney Radtke^157^, Francesca Ragona^81^, Mark I Rees^59, 158^, Sylvain Rhelms^159, 160^, Antonella Riva^4, 5^, Felix Rosenow^118, 119, 121^, Philippe Ryvlin^161^, Anni Saarela^105, 106^, Lynette G Sadleir^117^, Josemir W Sander^11, 16, 140^, Thomas Sander^2, 162^, Marcello Scala^4, 5^, Theresa Scattergood^163^, Steven C Schachter^164^, Christoph J Schankin^165, 166^, Ingrid E Scheffer^30, 31, 167, 168^, Bettina Schmitz^169^, Susanne Schoch^170^, Susanne Schubert-Bast^119, 121^, Andreas Schulze-Bonhage^171^, Paolo Scudieri^4, 5^, Beth R Sheidley^155^, Jerry J Shih^172^, Graeme J Sills^173^, Sanjay M Sisodiya^11, 16^, Michael C Smith^174^, Philip E Smith^175^, Anja C M Sonsma^114^, Doug Speed^176^, Michael R Sperling^177^, Bernhard J Steinhoff^21, 178^, Ulrich Stephani^95^, Remi Stevelink^114^, Carlotta Stipa^33^, Pasquale Striano^4, 5^, Hans Stroink^179^, Rainer Surges^24^, Toshimitsu Suzuki^180, 181^, K Meng Tan^6^, R.S.Taneja^1^, Erik Taubøll^97^, Liu Lin Thio^182^, G Neil Thomas^183^, Rhys H Thomas^184, 185^, Paolo Tinuper^34^, Marian Todaro^6, 7^, Pınar Topaloğlu^186^, Meng-Han Tsai^187-189^, Birute Tumiene^190, 191^, Dilsad Turkdogan^192^, Luc Valton^193^, Andreas van Baalen^95^, Annalisa Vetro^86^, Eileen P G Vining^194^, Randi von Wrede^24^, Ryan G Wagner^195, 196^, Yvonne Weber^197^, Sarah Weckhuysen^198-200^, Judith Weisenberg^182^, Michael Weller^201^, Peter Widdess-Walsh^47, 66^, Stefan Wolking^197^, Kazuhiro Yamakawa^180, 181^, Wanling Yang^134^, Zuhal Yapıcı^186^, Emrah Yücesan^202^, Felix Zahnert^118^, Federico Zara^4, 5^, Wei Zhou^51, 53, 54, 203^, Fritz Zimprich^125^, Gábor Zsurka^24, 128^, Quratulain Zulfiqar Ali^8^

1. Department of Neurology, Vanderbilt University Medical Center, Nashville, TN, USA.

2. Cologne Center for Genomics (CCG), University of Cologne, Faculty of Medicine and University Hospital Cologne, 50931 Cologne, Germany.

3. Tel-Aviv University Sackler Faculty of Medicine, Ramat Aviv 69978, Israel.

4. Department of Neurosciences, Rehabilitation, Ophthalmology, Genetics, Maternal and Child Health, University of Genova, Genova, Italy.

5. IRCCS Istituto Giannina Gaslini, Genova, Italy.

6. Department of Medicine, University of Melbourne, Royal Melbourne Hospital, Parkville 3050, Australia.

7. Department of Neuroscience, The School of Translational Medicine, Alfred Health, Monash University, Melbourne 3004, Australia.

8. Adult Genetic Epilepsy Program, University of Toronto, Toronto, ON, Canada.

9. Institute for Biomedical Research and Innovation, National Research Council, Cosenza, Italy.

10. St George's University Hospital NHS Foundation Trust, London, UK.

11. Department of Clinical and Experimental Epilepsy, UCL Queen Square Institute of Neurology, London WC1N 3BG, UK.

12. Genetics and Gene Regulation Division, The Walter and Eliza Hall Institute of Medical Research, Parkville 3052, Australia. .

13. Department of Biology, University of Melbourne, Parkville 3010, Australia. .

14. School of Mathematics and Statistics, University of Melbourne, Parkville 3010, Australia.

15. Swansea University Medical School, Swansea University, Swansea, Wales, UK.

16. Chalfont Centre for Epilepsy, Chalfont-St-Peter, Buckinghamshire SL9 0RJ, UK. .

17. Neuroscience and Medical Genetics Department, Meyer Children's Hospital IRCCS, Florence, Italy.

18. Department of NEUROFARBA, University of Florence, Florence, Italy.

19. University Health Network, University of Toronto, Toronto, ON, Canada.

20. APHM, Timone Hospital, Epileptology and Cerebral Rhythmology, Aix Marseille Univ, INSERM, INS, Inst Neurosci Syst, Marseille, France.

21. Epilepsy Center Kork, Kehl-Kork, Germany.

22. Medical Faculty of the University of Freiburg, Freiburg 79085, Germany.

23. Department of Psychiatry, The University of Hong Kong, Pokulam, Hong Kong.

24. Department of Epileptology, University of Bonn Medical Centre, Bonn 53127, Germany.

25. Department of Neurology, EMAR Medical Center, Istanbul, Turkey.

26. Department of Neurology, Istanbul Faculty of Medicine, Istanbul University, Istanbul, Turkey.

27. Department of Genetics, Aziz Sancar Institute of Experimental Medicine, Istanbul University, Istanbul, Turkey.

28. Section for Translational Epilepsy Research, Department of Neuropathology, University of Bonn Medical Center, Bonn 53105, Germany.

29. Department of Neurology, University of Ulm, Ulm 89081, Germany.

30. Epilepsy Research Centre, University of Melbourne, Austin Health, Heidelberg 3084, Australia.

31. Bladin-Berkovic Comprehensive Epilepsy Program, Austin Health, Heidelberg, Victoria, Australia.

32. Department of Neurology, American University of Beirut Medical Center, Beirut, Lebanon.

33. IRCCS Istituto delle Scienze Neurologiche di Bologna, Full Member of the ERN-EpiCARE, Bologna, Italy.

34. Department of Biomedical and Neuromotor Sciences, University of Bologna, Bologna, Italy.

35. Department of Pediatric Neurology, Dr von Hauner Children's Hospital, Ludwig Maximilians University, Munchen, Germany.

36. Epilepsy Center Munich, Munich, Germany.

37. Department of Neurology and Epileptology, Hertie Institute for Clinical Brain Research, University of Tübingen, Tübingen 72076, Germany.

38. Center for Applied Genomics, The Children's Hospital of Philadelphia, Philadelphia, PA 19104, USA.

39. Quantinuum Research LLC, Wayne, PA 19087, USA.

40. National Human Genome Research Institute, National Institutes of Health, Bethesda, MD 20892, USA.

41. Department of Biomedical Sciences, Cooper Medical School of Rowan University Camden, NJ 08103, USA.

42. Department of Neurology, Thomas Jefferson University Hospital, Philadelphia, PA 19107, USA.

43. Genomic Medicine Institute, Lerner Research Institute, Cleveland Clinic, Cleveland, OH 44195, USA.

44. Cleveland Clinic Epilepsy Center, Neurological Institute, Cleveland Clinic, Cleveland, OH 44195, USA.

45. Department of Neurology, Neurological Institute, Cleveland Clinic, Cleveland, OH 44195, USA.

46. School of Pharmacy and Biomolecular Sciences, The Royal College of Surgeons in Ireland, Dublin, Ireland.

47. The FutureNeuro Research Centre, Dublin, Ireland.

48. Belfast Health and Social Care Trust, Belfast BT9 7AB, UK.

49. Integrated Diagnostics for Epilepsy, Fondazione IRCCS Istituto Neurologico C. Besta, Milan, Italy.

50. Unit of Genetics of Neurodegenerative and Metabolic Diseases, Fondazione IRCCS Istituto Neurologico Carlo Besta, Milan, Italy.

51. Stanley Center for Psychiatric Research, Broad Institute of Harvard and M.I.T., Cambridge, MA 02142, USA.

52. Hôpital Lariboisière, Dept of Neurosurgery-Paris-Cité University, Paris, France.

53. Analytic and Translational Genetics Unit, Department of Medicine, Massachusetts General Hospital and Harvard Medical School, Boston, MA 02114, USA.

54. Program in Medical and Population Genetics, Broad Institute of MIT and Harvard, Cambridge, MA, USA.

55. Department of Epidemiology and Preventive Medicine, School of Public Health, Gray Faculty of Medical and Health Sciences, Tel Aviv University, Tel Aviv 6997801, Israel.

56. Department of Pharmacology and Pharmacy, The University of Hong Kong, Pokfulam, Hong Kong.

57. Department of Pediatric Neurology, Chang Gung Memorial Hospital, Linkou Branch, and College of Medicine, Chang Gung University, Taoyuan, Taiwan.

58. Kids Research, Children’s Hospital at Westmead Clinical School, Faculty of Medicine and Health, University of Sydney, Sydney, New South Wales, Australia.

59. Neurology Research Group, Swansea University Medical School, Faculty of Medicine, Health & Life Science, Swansea University, SA2 8PP, UK.

60. Epilepsy Service, Department of Neurology Massachusetts General Hospital, Boston, MA, USA.

61. Department of Neuroscience, Reproductive and Odontostomatological Sciences, University Federico II, Naples 80131, Italy.

62. Division of Neurology, Children’s Hospital of Philadelphia, Philadelphia, 3401 Civic Center Blvd, Philadelphia, PA 19104, USA.

63. The Epilepsy NeuroGenetics Initiative (ENGIN), Children's Hospital of Philadelphia, Philadelphia, 3401 Civic Center Blvd, Philadelphia, PA 19104, USA.

64. Department of Neurosciences, Université de Montréal, Montréal, CA 26758, Canada.

65. Department of Neurology, Royal Victoria Hospital, Belfast Health and Social Care Trust, Grosvenor Road, Belfast BT12 6BA, UK.

66. Department of Neurology, Beaumont Hospital, Dublin D09 FT51, Ireland.

67. Department of Neurology, CUB Erasme Hospital, Hôpital Universitaire de Bruxelles (H.U.B.), Université Libre de Bruxelles (ULB), Bruxelles 1070, Belgium.

68. NYU Langone Health, New York, NY, USA.

69. Neurology Department, St. James’s Hospital, Dublin D03 VX82, Ireland.

70. Academic Unit of Neurology, School of Medicine, Trinity College Dublin, Dublin 2, Ireland.

71. The FutureNeuro Research Centre for Rare and Chronic Neurological Diseases, Dublin, Ireland.

72. Beta Neurology, 53277 Bonn, Germany.

73. Department of Neurology, University of Pennsylvania, Perelman School of Medicine, Philadelphia, PA, 19104 USA.

74. Department of General Practice and Primary Health Care, University of Helsinki and Helsinki University Hospital, Helsinki 0014, Finland.

75. Human Genetics Training Program, Vanderbilt University, Nashville, TN, USA.

76. Institute of Health Data Analytics and Statistics, College of Public Health, National Taiwan University, Taipei 100, Taiwan.

77. Department of Pharmacology and Psychiatry, University of Pennsylvania Perlman School of Medicine, Philadelphia, PA 19104, USA.

78. Department of Pediatrics and Neonatology, Medical University of Vienna, Vienna 1090, Austria; Center for Rare and Complex Epilepsies, Member of ERN EpiCARE.

79. Institute of Neurology, Department of Medical and Surgical Sciences, University “Magna Graecia”, Catanzaro, Italy.

80. NYU Grossman School of Medicine, New York City, NY 10016, USA.

81. Department of Pediatric Neuroscience, Fondazione IRCCS Istituto Neurologico Carlo Besta, Milan, Italy.

82. Department of Medical and Surgical Sciences, Neuroscience Research Center, Magna Graecia University, viale Europa, Catanzaro, Italy.

83. IRCCS Azienda Ospedaliero-Universitaria di Bologna, Medical Genetics Unit, Bologna, Italy.

84. Cincinnati Children's Hospital Medical Center, Cincinnati, Ohio, USA.

85. Department of Neurology, Baylor College of Medicine.

86. Pediatric Neurology, Neurogenetics and Neurobiology Unit and Laboratories, Children's Hospital A. Meyer, University of Florence, Italy.

87. Division of Human Genetics, Department of Pediatrics, The Perelman School of Medicine, University of Pennsylvania, Philadelphia, PA 19104, USA.

88. Life and Brain Center, University of Bonn Medical Center, Bonn 53127, Germany.

89. Luxembourg Centre for Systems Biomedicine, University of Luxembourg, Esch-sur-Alzette L-4362, Luxembourg.

90. Institute for Genomic Statistics and Bioinformatics, University of Bonn, Bonn, Germany.

91. Department of Neurology, University of California, San Francisco, CA 94143, USA.

92. Division of Pharmacotherapy and Experimental Therapeutics, Eshelman School of Pharmacy, University of North Carolina at Chapel Hill, Chapel Hill, NC, 27599, USA.

93. Department of Genetics, School of Medicine, University of North Carolina at Chapel Hill, Chapel Hill, NC, 27599, USA.

94. Institute of Clinical Molecular Biology, Christian-Albrechts-University of Kiel, University Hospital Schleswig Holstein, Kiel 24105, Germany. .

95. Department for children and youth medicine II, University Medical Center Schleswig-Holstein, Campus Kiel, Kiel university, 24105 Kiel, Germany.

96. Department of Biomedical and Health Informatics (DBHi), Children’s Hospital of Philadelphia, Philadelphia, PA, 19104 USA.

97. Department of Neurology, Oslo University Hospital - Rikshospitalet and Faculty of Medicine, University of Oslo, Oslo, Norway.

98. General Medical Research Center, School of Medicine, Fukuoka University, Japan.

99. Department of Neurology, University Hospital of Strasbourg, Strasbourg, France.

100. Swiss Epilepsy Center, Klinik Lengg, Zurich, Switzerland.

101. National Epilepsy Center, Shizuoka Institute of Epilepsy and Neurological Disorder, Shizuoka, Japan.

102. Department of Psychiatry and Applied Psychology, Institute of Mental Health University of Nottingham, Nottingham NG7 2TU, UK.

103. Center for computational life sciences, Cleveland Clinic Research, Cleveland, OH 44195 USA.

104. Department of Brain Sciences, Imperial College London, London SW7 2AZ, UK.

105. Kuopio Epilepsy Center, Pediatric Neurology, Kuopio University Hospital, Kuopio 70210, Finland.

106. Institute of Clinical Medicine, University of Eastern Finland, Kuopio 70210, Finland.

107. Department of Computational Biology and Medical Sciences, Graduate School of Frontier Sciences, the University of Tokyo, Tokyo, Japan.

108. Hereditary Research Lab, Bethlehem University, Bethlehem, Palestine.

109. Department of Statistical Genetics, Osaka University Graduate School of Medicine, Suita, Japan.

110. Department of Child Neurology, Medical School, Kocaeli University, Kocaeli, Turkey.

111. Neuroscience Unit, KEMRI-Wellcome Trust Research Programme, Kilifi, Kenya.

112. Department of Public Health, Pwani University, Kilifi, Kenya.

113. Department of Psychiatry, University of Oxford, Oxford, UK.

114. Department of Genetics, University Medical Center Utrecht, Utrecht 3584 CX, The Netherlands.

115. Department of Pediatrics, Showa University School of Medicine, Epilepsy Medical Center, Showa University Hospital, 1-5-8 Hatanodai, Shinagawa-ku, Tokyo 142-8555, Japan.

116. Department of Pharmacology and Toxicology, American University of Beirut Faculty of Medicine, Beirut, Lebanon.

117. Department of Paediatrics and Child Health, University of Otago, Wellington, New Zealand.

118. Epilepsy Center Hessen-Marburg, Department of Neurology, Philipps University Marburg, Marburg, Germany.

119. Goethe University Frankfurt, Epilepsy Center Frankfurt Rhine-Main, Center of Neurology and Neurosurgery, Frankfurt, Germany.

120. Departments of Clinical Neurosciences, Medical Genetics and Community Health Sciences, Hotchkiss Brain Institute & Alberta Children’s Hospital Research Institute, Cumming School of Medicine, University of Calgary, Calgary, Alberta, Canada.

121. LOEWE Center for Personalized Translational Epilepsy Research (CePTER), Goethe University Frankfurt, Germany.

122. Department of Pediatrics, Institute of Rehabilitation, Transition and Palliation of Neurologically ill Children, Paracelsus Medical University, Salzburg, Austria; Member of the ERN Epicare.

123. Center for Pediatric Neurology, Neurorehabilitation and Epileptology, Schoen-Clinic Vogtareuth, Vogtareuth, Germany.

124. Cyprus Institute of Neurology and Genetics, Nicosia, Cyprus.

125. Department of Neurology, Medical University of Vienna, Vienna 1090, Austria.

126. Yale School of Medicine, New Haven, CT 06510, USA.

127. Institute of Human Genetics, University of Leipzig Medical Center, Leipzig, Germany.

128. Institute of Experimental Epileptology and Cognition Research, Medical Faculty, University of Bonn, Bonn, Germany.

129. Bonifatius Hospital Lingen, Neuropediatrics Wilhelmstrasse 13, 49808 Lingen, Germany.

130. Department of Neurology, Hofstra-Northwell Medical School, New York, NY, USA.

131. Department of Medicine and Therapeutics, Chinese University of Hong Kong, Hong Kong, China.

132. Department of Biomedical and Dental Sciences, Morphological and Functional Images (BIOMORF), University of Messina, Messina, Italy.

133. Luxembourg Institute of Health, Strassen, Luxembourg.

134. Department of Paediatrics and Adolescent Medicine, The University of Hong Kong, Hong Kong.

135. Neurocenter Suedwest, Boeblingen, Germany.

136. Department of Medical Genetics, Hospices Civils de Lyon and University of Lyon, Lyon, France.

137. Neurology Department, North Bristol Trust, Southmead Hospital, Bristol, BS10 5NB, UK.

138. Department of Health Technology and Informatics, The Hong Kong Polytechnic University, Hung Hum, Hong Kong.

139. Neuroscience Department, Janssen Research & Development, LLC, 1125 Trenton-Harbourton Road, Titusville, NJ, 08560, USA.

140. Stichting Epilepsie Instellingen Nederland (SEIN), Heemstede 2103 SW, The Netherlands.

141. Department of Medical Genetics and Genomic Medicine, School of Medical Sciences, University of Campinas (UNICAMP), and the Brazilian Institute of Neuroscience and Neurotecnology; Campinas, SP, Brazil.

142. Department of Medicine, Tseung Kwan O Hospital, Hong Kong.

143. Department of Pharmacology and Therapeutics, University of Liverpool, Liverpool L69 3GL, UK.

144. Neurology, Feinberg School of Medicine, Northwestern University, Chicago, IL, USA.

145. Division of Intramural Population Health Research, Eunice Kennedy Shriver National Institute of Child Health and Human Development, National Institutes of Health, Bethesda, MD 20892, USA.

146. Danish Epilepsy Centre, Dianalund 4293, Denmark. .

147. Institute of Regional Health Services Research, University of Southern Denmark, Odense 5000, Denmark.

148. Epilepsy Center for Children, University Hospital Ruppin-Brandenburg, Brandenburg Medical School, 16816 Neuruppin, Germany.

149. Pediatric Neurology, University of Giessen, Germany.

150. University Hospital Cologne, Cologne, Germany.

151. Laboratory for Systems Genetics, RIKEN Center for Integrative Medical Sciences, Yokohama, Japan.

152. Istanbul University-Cerrahpaşa, Cerrahpaşa Medical Faculty, Department of Neurology, Istanbul, Turkey.

153. Department of Neurology, Morriston Hospital, Swansea Bay University Bay Health Board, Swansea, Wales, UK.

154. IRCCS Azienda Ospedaliero-Universitaria di Bologna, Computational Genomics Unit, Bologna, Italy.

155. Department of Neurology, Boston Children's Hospital, Boston, MA, USA.

156. Department of Neurology, Gardner Neuroscience Institute, University of Cincinnati Medical Center, Cincinnati, OH 45220, USA.

157. Department of Neurology, Duke University School of Medicine, Durham, NC 27710, USA.

158. Faculty of Medicine & Health, University of Sydney, Sydney, New South Wales, Australia.

159. Department of Functional Neurology and Epileptology, Hospices Civils de Lyon and University of Lyon, France.

160. Lyon’s Neuroscience Research Center, INSERM U1028 / CNRS UMR 5292, Lyon, France.

161. Department of Clinical Neurosciences, Centre Hospitalo-Universitaire Vaudois, Lausanne, Switzerland.

162. Department of Neurology, Charité Universitaetsmedizin Berlin, Campus Virchow-Clinic, Berlin 13353, Germany.

163. Division of Endocrinology, Diabetes, and Metabolism, Perelman School of Medicine at the University of Pennsylvania, Philadelphia, PA, USA.

164. Departments of Neurology, Beth Israel Deaconess Medical Center, Massachusetts General Hospital, and Harvard Medical School, Boston, MA 02215, USA.

165. Department of Neurology, Inselspital, Bern University Hospital, University of Bern, Bern 3010, Switzerland.

166. Centre for Migraine and Headache, Bellevue Medical Group, Zurich, Switzerland.

167. Department of Neurology, Royal Children's Hospital, Parkville 3052, Australia.

168. Florey Institute of Neuroscience and Mental Health, Heidelberg, Victoria, Australia.

169. Department of Neurology, Stroke Unit and Epilepsy Center, Vivantes Humboldt-Klinikum Berlin and Charité Universitätsmedizin Berlin, Germany.

170. Institute of Cellular Neurosciences II, University of Bonn Medical Center, Bonn 53127, Germany.

171. Department of Epileptology, University Hospital Freiburg, Freiburg, Germany.

172. Department of Neurosciences, University of California, San Diego, La Jolla, CA 92037, USA.

173. School of Cardiovascular & Metabolic Health, University of Glasgow, Glasgow G12 8QQ, UK.

174. Rush University Medical Center, Chicago, IL 60612, USA. .

175. Department of Neurology, Alan Richens Epilepsy Unit, University Hospital of Wales, Cardiff CF14 4XW, UK. .

176. Center for Quantitative Genetics and Genomics, Aarhus University, Denmark.

177. Department of Neurology and Comprehensive Epilepsy Center, Thomas Jefferson University, Philadelphia, PA 19107, USA. .

178. Clinic for Neurology and Neurophysiology, University of Freiburg, Freiburg, Germany.

179. CWZ Hospital, 6532 SZ Nijmegen, The Netherlands.

180. Department of Neurodevelopmental Disorder Genetics, Institute of Brain Science, Nagoya City University Graduate School of Medical Science, Nagoya, Aichi, Japan.

181. Laboratory for Neurogenetics, RIKEN Center for Brain Science, Wako, Saitama, Japan.

182. Department of Neurology, Washington University School of Medicine, St. Louis, MO 63110, USA. .

183. Institute for Applied Health Research, University of Birmingham, Birmingham B15 2TT, UK. .

184. Translational and Clinical Research Institute, Newcastle University, Newcastle Upon Tyne, UK.

185. Department of Clinical Neurosciences, Newcastle Upon Tyne Hospitals NHS Foundation Trust, Newcastle Upon Tyne, UK.

186. Department of Child Neurology, Istanbul Faculty of Medicine, Istanbul University, Istanbul, Turkey.

187. Department of Neurology, Kaohsiung Chang Gung Memorial Hospital, Kaohsiung, Taiwan.

188. Doctoral Program of Clinical and Experimental Medicine, College of Medicine, National Sun Yat-sen University, Taiwan.

189. School of Medicine, College of Medicine, Chang Gung University, Taiwan.

190. Centre for Medical Genetics, Vilnius University Hospital Santaros Klinikos, Vilnius, Lithuania.

191. Institute of Biomedical Sciences, Faculty of Medicine, Vilnius University, Vilnius, Lithuania.

192. Department of Child Neurology, Medical School, Marmara University, Istanbul, Turkey.

193. Epilepsy Unit, Department of Neurology, Brain and Cognition Research Center - CerCo, CNRS, UMR5549, University Hospital and University of Toulouse, Paul Sabatier University, Toulouse, France.

194. Department of Neurology, The Johns Hopkins University School of Medicine, Baltimore, MD 21287, USA.

195. MRC/Wits Rural Public Health & Health Transitions Research Unit (Agincourt), School of Public Health, Faculty of Health Sciences, University of the Witwatersrand, Johannesburg, South Africa.

196. Barrow Neurological Institute, Department of Neurology, Phoenix, AZ, USA.

197. Section of Epileptology, Department of Neurology, RWTH University of Aachen, Aachen 52074, Germany.

198. VIB Center for Molecular Neurology, VIB, Antwerp, Belgium.

199. Department of Neurology, Antwerp University Hospital, Edegem 2650, Belgium.

200. Translational Neurosciences, Faculty of Medicine and Health Science, University of Antwerp, Antwerp, Belgium.

201. Department of Neurology, University Hospital and University of Zurich, Zurich, Switzerland.

202. Istanbul University-Cerrahpasa, Institute of Neurological Sciences, Department of Neurogenetics, Istanbul, Türkiye.

203. Psychiatric & Neurodevelopmental Genetics Unit, Department of Psychiatry, Massachusetts General Hospital and Harvard Medical School, Boston, MA 02114, USA.
